# Supplementation of Juçara Berry (*Euterpe edulis* Mart.) Modulates Epigenetic Markers in Monocytes from Obese Adults: A Double-Blind Randomized Trial

**DOI:** 10.3390/nu10121899

**Published:** 2018-12-03

**Authors:** Aline Boveto Santamarina, Giovana Jamar, Laís Vales Mennitti, Helena de Cássia César, Verdiana Vera de Rosso, José Ronnie Vasconcelos, Lila Missae Oyama, Luciana Pellegrini Pisani

**Affiliations:** 1 Programa de Pós-Graduação Interdisciplinar em Ciências da Saúde, Universidade Federal de São Paulo, Santos 11015-020, Brazil; alinesantamarina@gmail.com (A.B.S.); gi.jamar@gmail.com (G.J.); laisvmennitti@hotmail.com (L.V.M.); helenacesarbr@gmail.com (H.d.C.C.); 2 Departamento de Biociências, Universidade Federal de São Paulo, Santos 11015-020, Brazil; veriderosso@yahoo.com (V.V.d.R.); jrcvasconcelos@gmail.com (J.R.V.); 3 Departamento de Fisiologia, Universidade Federal de São Paulo, São Paulo 04023-062, Brazil; lmoyama@gmail.com; 4 Laboratório de Nutrição e Fisiologia Endócrina (LaNFE), Departamento de Biociências, Instituto de Saúde e Sociedade, Universidade Federal de São Paulo, Rua Silva Jardim, 136, Térreo, Vila Mathias, Santos, São Paulo 11015-020, Brazil

**Keywords:** obesity, epigenetics, fatty acids, monounsaturated fatty acid, juçara, monocytes

## Abstract

Nutrigenomics is an emerging field in obesity since epigenetic markers can be modified by environmental factors including diet. Considering juçara composition—rich in anthocyanins, monounsaturated fatty acids (MUFAs) and fibers—it has the potential for epigenetic modulation. We evaluated the juçara supplementation modulating the serum fatty acids profile and epigenetic markers in monocytes of adult obese humans. It was a randomized double-blind, controlled trial with 27 obese (Body mass index between 30.0 and 39.9 kg/m^2^) participants of both genders aged from 31 to 59 years, divided into juçara group (5 g juçara freeze-dried pulp) or placebo group (5 g of maltodextrin) for 6 weeks. Before and after supplementation, blood samples were collected. The serum and monocytes cells obtained were cultured and stimulated with lipopolysaccharides as proinflammatory stimulus. After 24 h of incubation, the cells and supernatants were collected and analyzed. Juçara improved the serum fatty acids profile on unsaturated fatty acids levels. The epigenetic markers evaluated were improved post-treatment. Also, the methylated DNA level was increased after treatment. We find that juçara supplementation is a predictor of methyl CpG binding proteins 2 (MeCP2) in monocytes. Concluding, juçara supplementation improved the serum fatty acids profile, modulating the epigenetic markers in monocytes from obese individuals.

## 1. Introduction

Obesity has a multifactorial etiology involving genetic and environmental factors [1], favoring the development of chronic non-communicable diseases [2,3]. In this sense, the nutrigenomics is an emerging field of research mainly because epigenetic markers can exhibit plasticity throughout the life course, even in varying degrees, and can be modified by environmental factors including diet [4].

Epigenetic is based on reversible changes in gene expression, which do not involve modifications in the nucleotide sequence of DNA. Among the major epigenetic regulators of gene transcription silencing and activation are alterations in DNA methylation, cytosine residues of CpG dinucleotides, covalent modifications in histone lysine residues (methylation and acetylation) and variations in chromatin structure [5,6,7].

The process involved in transcriptional silencing modulates gene expression through DNA methylation requiring the recruitment of DNA methyltransferase enzymes (DNMT1, DNMT3a, and DNMT3b), methyl CpG binding proteins (MeCP), histone deacetylases (HDAC) and histone methyltransferases (HMT) [8]. However, studies investigating the methylation of DNA histones in monocytes in obesity are still restricted [9,10,11]. There is evidence that DNMT enzymes are rhythmically recruited by promoters of chemokine binding genes into monocytes, which results in the silencing of cyclic genes by controlling the numbers of proinflammatory monocytes [12,13].

In recent years, emerging reports have provided evidence that phytochemicals, such as anthocyanins, can exert their advantageous effects by targeting epigenetic mechanisms via regulation of specific epigenetic components such as DNA methyltransferases (DNMTs), histone deacetylases (HDACs), histone acetyltransferases (HATs) and small non-coding RNAs (miRNAs) modulating fatty acid β-oxidation genes in obesity [14]. Another nutrient able to modulate the epigenetic mechanisms is fatty acids. In vivo and in vitro studies have demonstrated that unsaturated fatty acids are able to reduce the activity of pro-inflammatory genes *in loci* as well as improve the mitochondrial energy expenditure [4,15]. Nonetheless, these mechanisms are still little elucidated in literature.

Juçara berry (*Euterpe edulis* Mart.)—which comes from a palm tree—is commonly found in the Atlantic Rain Forest. It is a small and round fruit with a color which evolves from green to black during the ripening process similar to *Euterpe oleracea* fruits used to produce açaí. Juçara pulp has been most consumed in the form of juice and later as an ingredient in many foods such as ice cream, sweets or beverages [16].

Besides, recent studies have demonstrated that juçara has high nutritional value, health benefits and could be a potential epigenetic modulator to be explored [17,18,19,20,21]. Its composition is rich in phytochemicals, mainly the anthocyanins cyanidin 3-rutinoside and monounsaturated fatty acids (MUFA) as oleic fatty acid (C18:1n9) as well as dietary fibers. Considering this, we proposed to evaluate the alterations of serum fatty acid profile and the capacity by juçara pulp in modulating the epigenetic markers in monocytes of adult obese humans.

## 2. Materials and Methods

### 2.1. Experimental Design

It was a randomized double-blind study, and placebo-controlled. After starting the clinical study, there were no changes in the methodology. The randomization was performed by the software (www.random.org) as a blind spot for the study, and the volunteers’ allocation was made at the moment they agreed to participate in the study, after having completed the first evaluations.

The sample size enrolled was based on a calculation determined using the G*Power software [22]. The test F family was chosen based on the analysis used to assess statistical parameters for the primary outcome. We considered the value of effect size as 0.8, power (1-β err prob) was set at 95%, and α at 0.05. After calculating, it was added a safety margin of 20%, totalizing a sample of 28 volunteers. For the western blotting and RT-PCR (revese transcription polymerase chain reaction) analyses, it was calculated a sub-sample following the steps above cited, considering previously reported means and standard errors of the mean [10,23,24,25,26,27,28]—this calculation resulted in a sample size of at least 4 per group for a reliable statistic result.

### 2.2. Participants

Twenty-seven obese individuals (Body mass index—BMI ≥ 30.0 ≤ 39.9 kg/m^2^) [29] of both genders, aged from 31 to 59 were addressed in this study. This clinical trial was previously submitted and, approved by the Ethics Committee of the Universidade Federal de São Paulo—Plataforma Brasil database (CEP-UNIFESP nº 0319/2017). It also meets the guidelines set in the International Declaration of Helsinki and all participants provided the written informed consent.

The following exclusion criteria were adopted: BMI out of the proposed range; infectious illness and/or severe chronic diseases; use of medication that interferes in the inflammatory cascade, lipid metabolism, and food consumption; alcohol and/or drug abuse; pregnancy or menopause.

### 2.3. Anthropometric Measurements

Anthropometric data were evaluated before and after a 6-week supplementation. To obtain body mass, the individuals were wearing light clothes and no shoes on an electronic scale of 0.1 kg precision (Toledo^®^, São Paulo, Brazil). The height was measured in erect posture in contact with the measurement scale, positioning the head horizontally—as Frankfurt’s plane—with a standardized wall-mounted stadiometer (Sanny^®^, Standard, São Paulo, Brazil). Waist circumference was measured at the midpoint between the iliac crest and the last rib after a natural expiration [30], considering the cut-off points proposed by the International Diabetes Federation—IDF [31].

Body mass index (BMI)—body mass (kg)/height (m^2^)—was calculated and classified as recommended by the World Health Organization. The waist-to-height ratio (W/H) was also calculated using a 0.5 cutoff point [32].

### 2.4. Supplementation

The supplementation was continued for 6 weeks, and the volunteers received the portioned sachets weekly, containing 5 g of the freeze-dried juçara pulp or 5 g of flavored maltodextrin, as a placebo. The volunteers were instructed to consume one sachet/day in the morning inserted in their usual breakfast habits. The participants were also instructed to keep the same lifestyle and diet habits during the supplementation period.

Each juçara berry weighs approximately 1 g with 1–1.5 cm of diameter. The juicy thin mesocarp and pericarp have been used to obtain the pulp. Five grams of freeze-dried juçara pulp per day is equivalent to the consumption of 50 g of fresh juçara pulp, which could be consumed in the commercial form of the pulp as the home measure of an ice cream ball [16].

The fruit dosage was based on previous studies considering the anthocyanins content of the pulp as well as on our previously dose-response test to offer a physiological consumption which exerts beneficial effects [19,20,21,33]. The juçara (*Euterpe edulis* Mart.) composition was previously summarized by Santamarina et al. [21] and presented in Table 1.

### 2.5. Dietary Intake

The dietary intake was evaluated before and after the supplementation period, through a self-reported 3-day Dietary Record. To know the participant’s dietary habits, a weekend day self-report was included in the 3-day Dietary Record. The data obtained were adjusted and the average of these records was analyzed using the specific software Avanutri 4.0 Revolution (Avanutri & Nutrition Services and Informatics Inc., Três Rios, RJ, Brazil).

### 2.6. Sample Collection

Blood samples (25 mL) were collected from the antecubital vein—in tubes with 125 IU of heparin sodium—before and after the supplementation period, after 12-h fasting. 5 mL of the blood was centrifuged at 690× *g* for 15 min at 4 °C to obtain serum, while the remaining 20 mL were used to isolate mononuclear cells. The peripheral blood mononuclear cells (PBMCs) were obtained by centrifugation at 400× *g* at room temperature for 30 min with Histopaque 1077 and Histopaque 1119. To separate monocytes from lymphocytes, a cell culture was done. After incubation, monocytes were adhered to the cell culture plate, while the lymphocytes were dispersed in the cell culture medium, which was discarded. The monocytes adhered were incubated at 37 °C with or without lipopolysaccharides (LPS) in RPMI-1640 medium supplemented with fetal bovine serum and antibiotics (2.5 mg/mL streptomycin and 2.5 IU/mL penicillin). After 24-h incubation, the supernatants and monocytes were collected to carry out the next experimental analyses. Sample pooling was not employed in any analyzes of this study.

### 2.7. Serum Fatty Acids Analyses by GC/FID

The serum free fatty acid profile was analyzed by gas chromatography (Varian GC 3900) coupled in flame ionization detection (FID) with a CP-8410 autosampler (Walnut Creek, CA, USA). The formation of fatty acids methyl esters (FAMEs) was performed with acetyl chloride (5% HCl in methanol) [35]. Samples were analyzed on a capillary column (CP Wax 52 CB, Varian, Lake Forest, CA, USA); 0.25 µm thickness, inside diameter 0.25 mm and 30 m length). Hydrogen was used as a carrier gas at a linear velocity of 22 cm/s. The temperature program was 170 °C for 1 min, followed by 2.5 °C/min of the increase until 240 °C and a final hold time of 5 min. The temperatures used were 250 and 260 °C for injector and FID, respectively. Fatty acids were identified by comparing the retention time using a known standard of FAMEs. (Supelco, 37 components; Sigma–Aldrich; Mixture, Me93, Larodan and Qualmix, PUFA fish M, Menhaden Oil, Larodan). Data were expressed as percentages of total fatty acids.

### 2.8. Total DNA Methylation and HDAC Enzymatic Activity

The nuclear extraction of mononuclear cells was performed with the EpiQuik Nuclear Extraction kit, 100 tests. Global DNA methylation and HDAC enzyme activity were performed in duplicate analyzes, using the monocyte nuclear extract with the specific kits MethylFlash Methylated DNA 5-mC Quantification Kit (Colorimetric) and Epigenase HDAC Activity/Inhibition Direct Assay Kit (Colorimetric) 96 tests (EPIGENTEK—USA) respectively. For both kits, all steps followed the recommendations of the manufacturer present in the protocol accompanying the product.

### 2.9. RNA Extraction and RT-PCR

The monocytes total RNA extraction was performed with Trizol Reagent^®^ (Thermo Fisher Scientific, Waltham, MA, USA) following the manufacturer’s recommendations. RNA concentration was measured by NanoDrop ND-2000 (NanoDrop Technologies Inc., Wilmington, DE, USA). RNA purity was stimated by the 260/280 nm ratio, showing values between 1.8 and 2.0. Two micrograms of total RNA samples were reverse transcribed using a High-capacity cDNA reverse transcription kit (Thermo Fisher Scientific Waltham, MA, USA), to synthesize complementary DNA (cDNA). The specific primers used were presented in Table 2.

Relative mRNA levels were assessed in duplicates by RT-PCR using SYBR green PCR master mix in a QuantStudioTM 7 Flex (Thermo Fisher Scientific). The HPRT (Hypoxanthine phosphoribosyltransferase) gene level was used as housekeeping. Results were obtained using the Sequence Detector software (Thermo Fisher Scientific) and were expressed as the relative increase using the method of 2^−ΔΔCt^ previously described by Livak and Schmittgen [36].

### 2.10. Western Blotting

Monocyte cell pellets were eluted and incubated in specific lysis buffer for 30 min. The sample was centrifuged at 20,800× *g* for 40 min at 4 °C, and the supernatant collected. The total protein concentration was determined in duplicates by Bradford reagent (LGC Laboratories, Inc., Cotia, SP, Brazil) and normalized to 50 µg per sample. The 10% SDS—polyacrylamide gel was performed to separate protein samples by electrophoresis and transferred to nitrocellulose membranes (Bio-Rad Laboratories Inc.). The membranes were blocked overnight with 1% bovine serum albumin solution at room temperature. The membranes were incubated overnight with the following primary antibodies: DNMT3a (sc-20703); DNMT3b (sc-20704) (Santa Cruz Biotechnology, Inc., Santa Cruz, CA, USA) DNMT1 (ab13537); MeCP2 (ab2829) (Abcam, Cambridge, UK). The β-actin (ab6276; Abcam, Cambridge, UK) was used as housekeeping. The specific horseradish peroxidase-conjugated secondary antibodies were incubated for 1 h at room temperature. Bands were visualized using enhanced chemiluminescence scanned using Alliance 4.7 equipment (Cambridge, UK) after adding ECL reagent (Bio-Rad Laboratories, Inc, Hercules, CA, USA). Scion Image (Scion Image-Release Beta 3b; NIH, Frederick, MD, USA) quantified the bands’ intensity.

### 2.11. Statistical Analyzes

Data obtained were submitted to the Grubb’s test for detecting outliers. The quality tests used were Shapiro–Wilk for normality, and Levene’s test for homogeneity. For the parametric variables, the two-way analysis of variance (ANOVA) followed by Bonferroni post hoc test was used to compare the groups. Pearson correlation was used to verify associations between variables. Multiple linear regression was applied to determine the predictors of epigenetic markers. All statistical tests were performed in the PASW Statistics software version 22.0. The results were expressed as the mean ± standard error of the mean (S.E.M.) and the significance level adopted was *p* < 0.05.

## 3. Results

### 3.1. Dietary Intake and Anthropometric Measurements

By analyzing the 3-day self-reported Dietary Record it was possible to obtain the energy intake and proportion values of macronutrients consumed by the volunteers at the beginning and after the period of supplementation. There were no differences in dietary intake either between the experimental groups or between the evaluation periods (Table 3).

The anthropometric measures of body mass and waist circumference did not change between the randomized groups either before or after the intervention period. On the same way, the BMI and waist-to-height ratio did not differ between the groups proposed before or after the supplementation period. These anthropometric variables also allow us to state that both groups had similar levels of obesity as shown in Table 4.

### 3.2. Serum Fatty Acids Profile

Evaluating the profile of serum fatty acids in the volunteers, before and after the period of supplementation, it is noteworthy the improvement of fatty acids profile in juçara group. It is noticed the reduction of ΣSFA (sum of saturated fatty acids) detected in the serum of juçara group compared to the placebo group after supplementation (*p* = 0.032).

The ΣMUFA (sum of monounsaturated fatty acids) detected in the samples were increased in juçara group compared to placebo (*p* = 0.034), after the supplementation period. This change is mainly related to the C16:1n7 and C18:1n9 fatty acid increase (*p* = 0.031 and *p* = 0.049. respectively) after juçara intake. The ΣPUFA ω-3 (sum of polyunsaturated ω-3 fatty acids) was increased after juçara supplementation (*p* = 0.044). In addition, the ω-6/ω-3 ratio also improved with an increase in ω-3 levels in juçara group after treatment compared to its baseline measurement (*p* = 0.036). These results are shown in Table 5.

### 3.3. Epigenetic Markers Gene Expression

**Figure 1:**
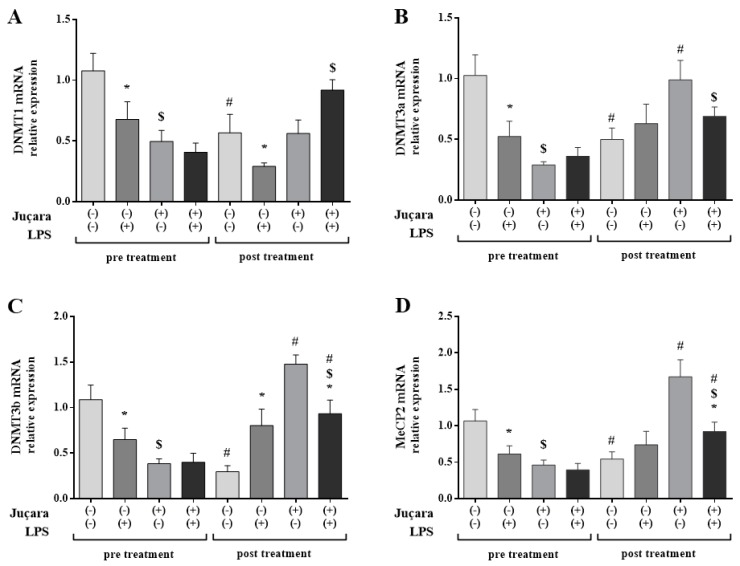
Monocyte epigenetic markers gene expression among groups before and after supplementation: (**A**) DNMT1; (**B**) DNMT3a; (**C**) DNMT3b; (**D**) MeCP2. The housekeeping used for all analyses was the HPRT gene expression. * without LPS versus with LPS; # baseline versus post-treatment; $ placebo versus juçara group. (n = 10 per group).

The DNMT1 mRNA levels were reduced in placebo group + LPS before (*p* = 0.012) and after (*p* = 0.013) treatment period. In pre-treatment, the juçara group without LPS had lower levels of DNMT1 mRNA compared to its placebo (*p* < 0.001). Furthermore, in the placebo without LPS DNMT1 was reduced after treatment in comparison of pre-treatment levels (*p* < 0.001). Post-treatment DNMT1 mRNA levels increased in juçara group + LPS compared to placebo (*p* < 0.001) as shown in Figure 1A.

In the pre-treatment the DNMT3a mRNA levels in the placebo group was reduced by the LPS stimulus (*p* = 0.004), also in pre-treatment the juçara was reduced compared to the placebo group both without LPS (*p* < 0.001). In the post-treatment, the juçara + LPS was higher than placebo + LPS (*p* = 0.003). In the post-treatment, the placebo without LPS group reduced DNMT3a mRNA levels in comparison of baseline (*p* = 0.001). In contrast, after treatment, the juçara group DNMT3a was increased compared to the pre-treatment measurements (*p* < 0.001) as demonstrated in Figure 1B.

DNMT3b mRNA levels in pre-treatment were decreased in the placebo group with LPS in comparison of placebo without LPS (*p* = 0.008). Also, in pre-treatment juçara without LPS had lower levels of DNMT3b mRNA (*p* < 0.001). After treatment, placebo group without LPS reduced DNMT3b compared to baseline (*p* = 0.041), also in post-treatment the LPS stimulus increased this gene levels compared to the placebo group without LPS (*p* < 0.001). The juçara supplementation increased the DNMT3b mRNA either with or without LPS in comparison of its pre-treatment measurements (*p* < 0.001 in both groups). Specifically, the post-treatment juçara group + LPS showed lower levels compared to juçara group without (*p* = 0.002); however, juçara + LPS demonstrated higher levels of DNMT3b than placebo + LPS (*p* < 0.001; Figure 1C).

The MeCP2 mRNA levels in pre-treatment were lower in placebo group + LPS compared to placebo without LPS (*p* = 0.028). Moreover, the pre-treatment juçara group had lower levels of MeCP2 in comparison of placebo both without LPS stimulus (*p* < 0.001). After the juçara supplementation, the placebo without LPS was reduced compared to baseline (*p* = 0.007). The post-treatment both juçara groups (with and without LPS) showed higher levels of MeCP2 mRNA compared to pre-treatment measurements (*p* < 0.001 and *p* = 0.017, respectively). Nonetheless, the post-treatment juçara + LPS demonstrated higher levels of MeCP2 than its placebo (*p* = 0.001), complementarily, juçara + LPS had lower levels in comparison of juçara without LPS (*p* < 0.001) as shown in Figure 1D.

### 3.4. Epigenetic Markers Protein Expression

**Figure 2:**
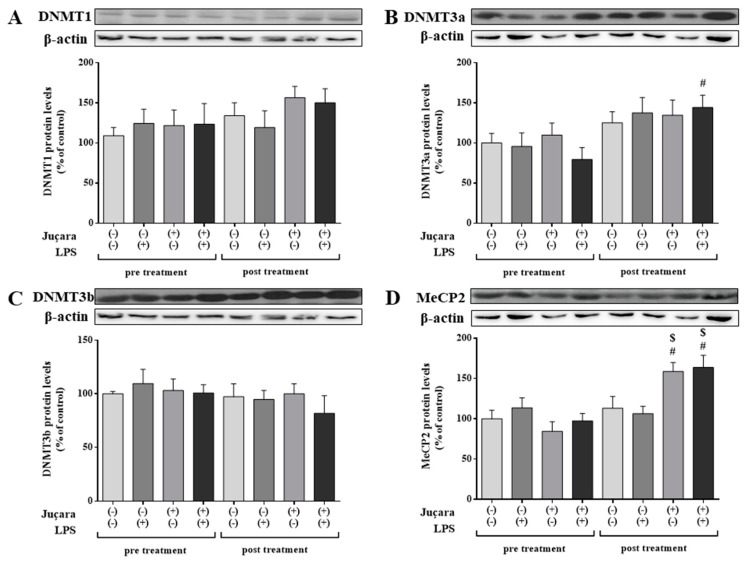
Monocyte epigenetic markers protein expression among groups before and after supplementation: (**A**) DNMT1; (**B**) DNMT3a; (**C**) DNMT3b; (**D**) MeCP2. The housekeeping used for all analyses was β-actin protein expression. # baseline versus post-treatment; $ placebo versus juçara group. (n = 10 per group).

The protein expression of DNMT3a was increased in juçara + LPS after treatment in comparison of baseline levels (*p* = 0.006; Figure 2B). Furthermore, MeCP2 expression of both juçara groups post-treatment was higher than pre-treatment evaluation (*p* < 0.001 for both groups with or without LPS). In addition, both juçara groups (without and with LPS) demonstrated increased expression of MeCP2 compared to the respective placebo groups after juçara supplementation (*p* = 0.010 and *p* = 0.002, respectively), showed in Figure 2D. The protein expression of DNMT1 and DNMT3b did not differ among the experimental groups either before or after treatment (Figure 2A,C).

### 3.5. HDAC Activity and Total DNA Methylation

**Figure 3:**
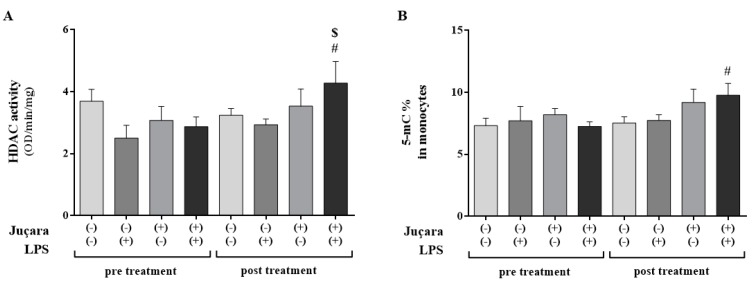
(**A**) Total HDAC activity and (**B**) methylated DNA levels in monocytes. # baseline versus post-treatment; $ placebo versus juçara group. (*n* = 10 per group).

After treatment, the total histones deacetylases (HDAC) activity was increased in juçara group + LPS versus its pre-treatment levels (*p* = 0.027) and compared to placebo group + LPS in post-treatment (*p* = 0.031) as shown in Figure 3A. Likewise, the DNA methylation was increased in the juçara group + LPS (*p* = 0.027) post-treatment in comparison of pre-treatment (Figure 3B).

### 3.6. Serum Fatty Acids Profile Predict Epigenetic Markers Gene Expression

After the Pearson correlation test of all fatty acids and epigenetic markers evaluated, we selected DNMT3a, MeCP2, ΣMUFA, C16:1n7 and C18:1n9 variables to apply the regression model. The multiple linear regression applied to determine that the juçara treatment (*p* < 0.001), as well as the serum concentration of oleic fatty acid (C18:1n9), were predictors of MeCP2 mRNA levels (*p* = 0.032) demonstrated in Table 6.

## 4. Discussion

Our results suggest that juçara pulp supplementation was able to improve the serum levels of monounsaturated fatty acids (MUFA), which predicted an epigenetic modulation in monocytes isolated from obese adults. It demonstrated that modifications in the serum fatty acid profile might regulate the expression of epigenetic markers and it could modulate inflammatory and metabolic pathways in obese adults. The lipids intake and fatty acids bloodstream profile are closely related to metabolic diseases development [37,38]. Our results demonstrate that there was no statistical difference in the composition of serum fatty acids during the initial collection of samples, demonstrating the proportional similarity between the randomization of the chosen groups. After the supplementation period, it was possible to observe a reduction of saturated fatty acids (SFA) in the juçara group. There was also an increase in the total circulating MUFA after treatment, mainly justified by the palmitoleic (C16:1n7) and oleic (C18:1n9). The increase of these serum fatty acids directly reflects in the juçara pulp propriety, since the fatty acids identified corresponded to those found in the composition [17]. Although there was no change in total PUFA levels, the amount of ω-3 PUFA increased and there was improve the ω-6/ω-3 ratio after the juçara pulp consumption. Recently, the role of the ω-6/ω-3 ratio in the diet upon cardiovascular and inflammatory diseases have been discussed. With food industrialization, there was an increase in the consumption of ω-6 fatty acids in detriment of ω-3 fatty acids intake. This process modified the usual ratio intake of approximately 1:1 to 2:1 for a ratio of 15:1 to 40:1 in the recent western dietary pattern. Currently, some studies proposed an optimal ω-6/ω-3 ratio to up to 4:1; however, there is no consensus on the consumption recommendation [39]. The importance of the ω-6/ω-3 relation is based on the competition by the action of the enzyme Δ-6 desaturase. High doses of linoleic acid (C18:2n6) reduced the enzymatic conversion of α-linolenic acid (C18:3n3) to eicosapentaenoic acid (EPA C20:5n3) and docosahexaenoic acid (DHA C22:6n3), favoring the increase of arachidonic acid (C20:4n6) [40]. In this sense, the improvement of ω-6/ω-3 ratio after supplementation may be occurred due to juçara lipids composition, and it possibly provided a reduction in the production of inflammatory markers from arachidonic acid.

These results demonstrate that the supplementation was able to improve the profile of circulating fatty acids in the serum, mainly by ω-3 PUFA and MUFA. The most popular source of MUFA is the olive oil. However, several nuts and fruits are sources of ω-9 fatty acids. It is also known that consumption of a diet rich in ω-9 fatty (3–23% dietary intake) acids exerts positive effects on the reduction of adiposity and cardiovascular risk as well as improvement of serum parameters—such as HDL-c and the LDL-c/HDL-c ratio [41,42,43,44].

Even with different amounts of MUFAs, whether the consumption or supplementation follow the DRI (Dietary Reference Intakes) for a balanced diet, the ω-9 fatty acids were able to exert positive effects [45]. Meanwhile, there is little discussion about the role of MUFAs modulating the immune system of obese individuals. In this sense, the consumption of ω-9 fatty acids as oleic acid (C18:1n9) may exert an anti-inflammatory effect and an improvement in insulin sensitivity. MUFA consumption is related to the improved production of anti-inflammatory cytokines and reduced pro-inflammatory markers in human and animal models of obesity [46,47,48]. Food supplementation of MUFA is also related to the reduction of infiltration of macrophages M1 (pro-inflammatory) and CD8 (+) T cells of adipose tissue even with high-fat diet intake [46]. Recent studies have also shown that the family of G protein-coupled receptors (GPR) such as GPR120 can be activated by isomers of ω-9 (C18:1n9) and ω-3 (C18:3n3, C20:5n3 and C22:6n3). The activation of these receptors may stimulate the production of GLP-1 (glucagon-like peptide-1) in addition to the signaling of anti-inflammatory mechanisms [49,50].

Polyphenols such as anthocyanins and unsaturated fatty acids provide health benefits associated with reduced risk of metabolic diseases. These nutrients exhibit a broad spectrum of biological activities, such as anti-oxidant; free radical-scavenger; anti-inflammatory; anti-carcinogenic; anti-viral; anti-bacterial; anti-thrombogenic; and anti-atherogenic activities [50]. The complexity and reversible feature of epigenetic modification in diseases offer promising targets for dietary nutrients intervention [4]. Considering the multifactorial features of obesity, understanding their regulatory role in epigenetic modifications may help in the prevention and treatment of the associated metabolic diseases.

Anthocyanins are well-known antioxidants for reactive oxygen species (ROS). Specifically cyanidin-3-glycoside—major anthocyanin in the juçara fruit—has a great ability to absorb oxygen radicals and consequently protect against oxidative stress. Anthocyanins have also been shown to inhibit the activation of (nuclear factor kappa B) NFκB via (mitogen activated protein kinases) MAPK and Cyclooxygenase (COX) pathway through DNA methylome changes. These biological activities of anthocyanins are closely related to the incidence of chronic diseases by reducing risk factors for cardiovascular disease, body weight and insulin resistance [51,52,53,54,55].

Evidence has shown that there are different methylation patterns of genes implicated in fatty acid β-oxidation in samples obtained from lean and severely obese women in response to lipid exposure. The peroxisome proliferator-activated receptor δ (PPAR-δ) was found to be differentially regulated in obesity due to different methylation patterns of the gene promoted by the DNMTs. Moreover, another gene epigenetically modulated was carnitine palmitoyltransferase 1B. CPT-1B is a protein responsible for transferring the long-chain fatty acids across the outer mitochondrial membrane provided by oleic fatty acids exposure due to DNA methylation, and histone acetylation [56]. Although these founds corroborate our hypothesis that the link between epigenetic modifications and obesity could be influenced by MUFAs and polyphenols prevenient from juçara, this mechanism remains as yet unestablished.

The human genome encodes five different DNMTs: DNMT1, DNMT2, DNMT3a, DNMT3b, and DNMT3L. Particularly, DNMT1, DNMT3a, and DNMT3b are known for catalyzing the addition of methylation marks to genomic DNA. The literature demonstrates that DNMT1 has been related to the maintenance of epigenetic imprinting occurred during the fetal development. The DNMT3a and DNMT3b are related to de novo methylation process, which occurs more frequently during the adulthood leading to modifications on the pre-existing epigenetic pattern [57]. It is interesting to note that the more pronounced changes in DNMTs expression were related to the juçara supplementation. In particular, the enzymes responsible for initiate the de novo methylation process recruiting the MeCP2 to start the changes in DNA methylation.

These results allow us to suggest that the juçara intake could modulate the lipid metabolism through epigenetic mechanisms since the epigenetic markers were changed after the supplementation period. To verify the association between the change in serum acid profile—mainly due to the increase of serum MUFAs after treatment—and the epigenetic modifications founded. The regression analysis was performed. It was possible to note that juçara supplementation was responsible for the increase in serum levels of C18:1n9, which seems to be a predictor of increased activation of MeCP2 in obese adult monocytes. To each 1 mg/dl of oleic fatty acid increased in the bloodstream, MeCP2 levels increased by 64%.

## 5. Conclusions

In conclusion, it was possible to perceive that juçara fruit supplementation modulated the serum fatty acids profile and could contribute for the reduction of the metabolic disease risk through the epigenetic modulation in obese individual monocytes—since the MUFAs predicted the DNA methylation promoter protein by MeCP2. Additionally, further analyzes of the epigenetic mechanisms are necessary to set the modulation mechanisms and to facilitate the establishment of strategies for the treatment and prevention of obesity and its comorbidities through epigenetics.

## Figures and Tables

**Table 1:** Juçara composition adapted from Santamarina et al. [21].

Juçara Pulp	Concentration in 100 g of Fresh Matter		References
	Mean	S.E.M.	
Carbohydrates (g)	28.3	3.5	[18]
Proteins (g)	6.0	0.3	[18]
Lipids (g)	29.2	0.9	[18]
Palmitic acid (%)	34.43	3.42	[17]
Stearic acid (%)	3.01	0.30	[17]
SAT (%)	37.44		[17]
Palmitoleic acid (%)	2.61	0.26	[17]
Oleic acid (%)	35.96	3.08	[17]
MUFA (%)	38.57		[17]
Linoleic acid (%)	19.18	1.89	[17]
Linolenic acid (%)	0.91	0.20	[17]
PUFA (%)	20.08		[17]
Fiber (g)	28.3	0.3	[18]
Ashes (g)	8.8	0.8	[18]
Energetic values (kcal)	400.0	23.9	[18]
Cyanidin 3-rutinoside (mg)	191.0	6.5	[34]
Cyanidin 3-glucoside (mg)	71.4	2.1	[34]
Total anthocyanins (mg)	262.4	8.6	[34]
Total phenolic compounds (mg)	415.1	22.3	[18]

SFA: total of dietary saturated fatty acids; MUFA: total of dietary monounsaturated fatty acids; PUFA: total of dietary polyunsaturated fatty acids.

**Table 2:** Primer sequences used for RT-PCR performance.

Target Genes	Sequences
DNMT1	5′-TCCTACGCCATGCCCAGTTTG-3′ (sense)
	5′-GAAGATGGGCGTCTCATCATCG-3′ (antisense)
DNMT3a	5′-GCCCATTCGATCTGGTGATTG-3′ (sense)
	5′-TCGTAAAGTCCCTTGCGGGC-3′ (antisense)
DNMT3b	5′-TGTGCAGAGTCCATTGCTGTAGGA-3′ (sense)
	5′-GCTTCCGCCAATCACCAAGTCAAA-3′ (antisense)
MeCP2	5’-CAGCTCCAACAGGATTCCATGGT-3′ (sense)
	5′-TGATGTCTCTGCTTTGCCTGCCT-3′ (antisense)
HPRT	5′-CCCTGGCGTCGTGATTAGTG-3′ (sense)
	5′-TCGAGCAAGACGTTCAGTCC-3′ (antisense)

**Table 3:** Dietary intake before and after the 6-week treatment period.

	Placebo (*n* = 14)		Juçara (*n* = 13)		*p*
	Initial	Final	Initial	Final	
Energy (kcal)	2051.82 ± 191.14	1960.98 ± 130.24	1997.01 ± 157.57	2107.45 ± 206.35	0.947
Carbohydrates (g)	258.58 ± 24.49	226.72 ± 16	253.93 ± 24.20	272.47 ± 28.86	0.740
Carbohydrates (%)	50.50 ± 1.54	46.65 ± 1.67	51.13 ± 1.60	51.65 ± 1.91	0.301
Protein (g)	82.85 ± 7.36	86.85 ± 8.48	81.17 ± 6.01	79.99 ± 9.33	0.710
Protein (%)	17.14 ± 1.03	22.15 ± 3.56	17.03 ± 1.10	15.17 ± 0.82	0.172
Lipids (g)	75.74 ± 8.96	73.86 ± 8.20	72.96 ± 6.38	77.51 ± 9.39	0.908
Lipids (%)	32.37 ± 1.25	33.73 ± 1.58	31.86 ± 1.01	33.09 ± 1.68	0.912
SFA	19.39 ± 3.05	22.07 ± 3.26	22.76 ± 2.21	22.63 ± 2.91	0.544
MUFA	18.75 ± 3.00	22.05 ± 3.44	18.53 ± 2.05	23.13 ± 3.33	0.912
PUFA	15.11 ± 2.07	14.21 ± 1.35	13.13 ± 1.97	14.67 ± 2.31	0.337

SFA: total of dietary saturated fatty acids; MUFA: total of dietary monounsaturated fatty acids; PUFA: total of dietary polyunsaturated fatty acids. These variables were compared by two-way ANOVA and Bonferroni post hoc test.

**Table 4:** Sample descriptive characteristics.

	Placebo (*n* = 14)		Juçara (*n* = 13)		*p*
	Initial	Final	Initial	Final	
Male/Female	06/08		05/08		-
Age (years)	45.07 ± 3.42		45.76 ± 2.58		-
Stature (m)	1.65 ± 0.03		1.66 ± 0.03		-
Body mass (kg)	92.23 ± 3.29	92.91 ± 3.57	96.42 ± 4.85	96.81 ± 5.09	0.700
BMI (kg/m^2^)	33.82 ± 0.71	34.06 ± 0.81	34.63 ± 1.20	34.76 ± 1.30	0.440
WC (cm)	102.70 ± 2.66	101.54 ± 2.38	104.59 ± 2.43	104.83 ± 2.85	0.608
W/H	0.62 ± 0.02	0.61 ± 0.01	0.63 ± 0.02	0.63 ± 0.01	0.809

WC: waist circumference; W/H: waist-to-height ratio. These variables were compared by two-way ANOVA and Bonferroni post hoc test.

**Table 5:** Serum fatty acids profile before and after supplementation for 6 weeks between experimental groups.

Fatty Acids (% of Total Identified)	Placebo (*n* = 14)		Juçara (*n* = 13)		*p*
	Initial	Final	Initial	Final	
ΣSFA	31.23 ± 2.08	35.00 ± 1.48	32.49 ± 1.03	30.21 ± 1.06 ^$^	*0.032*
C14:0	0.87 ± 0.13	1.42 ± 0.38	0.78 ± 0.05	0.86 ± 0.15	0.604
C16:0	19.91 ± 1.09	20.53 ± 1.43	21.20 ± 0.63	19.06 ± 0.87	0.494
C17:0	1.36 ± 0.28	1.78 ± 0.64	0.99 ± 0.32	0.98 ± 0.12	0.985
C18:0	7.06 ± 0.66	7.77 ± 0.44	7.71 ± 0.58	6.70 ± 0.61	0.680
C20:0	1.82 ± 0.57	2.34 ± 0.96	1.26 ± 0.48	1.70 ± 0.51	0.193
C24:0	0.73 ± 0.15	1.17 ± 0.22	0.63 ± 0.07	0.91 ± 0.23	0.223
ΣMUFA	27.91 ± 3.63	22.19 ± 1.23	26.17 ± 2.63	28.87 ± 2.78 ^$^	0.034
C16:1n7	2.97 ± 0.30	2.30 ± 0.16	2.76 ± 0.32	3.19 ± 0.35 ^$^	0.031
C18:1n9	23.29 ± 3.18	18.18 ± 1.07	21.73 ± 2.07	23.69 ± 2.29 ^$^	0.049
C18:1n7	1.80 ± 0.18	1.72 ± 0.11	1.99 ± 0.23	1.99 ± 0.20	0.359
ΣPUFA	40.91 ± 1.78	42.80 ± 1.91	41.34 ± 1.94	40.87 ± 3.40	0.834
C18:2n6	23.98 ± 0.96	24.28 ± 1.79	25.93 ± 1.51	20.10 ± 2.13	0.831
C18:3n6	2.10 ± 0.24	2.53 ± 0.37	1.70 ± 0.26	2.27 ± 0.36	0.658
C18:3n4	0.49 ± 0.06	0.50 ± 0.05	1.03 ± 0.41	0.48 ± 0.08	0.652
C18:4n3	0.14 ± 0.04	0.17 ± 0.03	0.31 ± 0.11	0.74 ± 0.33	0.783
C20:2n6	0.26 ± 0.03	0.28 ± 0.02	0.35 ± 0.11	1.32 ± 0.61	0.427
C20:3n6	1.13 ± 0.19	1.20 ± 0.16	1.87 ± 0.59	1.33 ± 0.23	0.466
C20:4n6	4.19 ± 0.57	5.93 ± 0.75	4.91 ± 0.67	4.90 ± 1.11	0.356
C20:3n3	1.06 ± 0.43	0.50 ± 0.16	0.67 ± 0.35	0.48 ± 0.31	0.552
C20:4n3	2.10 ± 0.54	2.50 ± 1.02	1.68 ± 0.51	3.08 ± 0.78	0.982
C20:5n3	0.87 ± 0.29	0.44 ± 0.09	0.45 ± 0.08	1.37 ± 0.77	0.496
C21:5n3	2.88 ± 0.67	2.64 ± 0.97	2.07 ± 0.61	3.07 ± 1.15	0.858
C22:4n6	0.19 ± 0.04	0.19 ± 0.03	0.26 ± 0.05	0.27 ± 0.11	0.259
C22:5n3	0.84 ± 0.46	0.51 ± 0.25	0.29 ± 0.04	0.52 ± 0.18	0.815
C22:6n3	0.69 ± 0.20	1.12 ± 0.25	0.88 ± 0.50	1.89 ± 0.99	0.537
ΣPUFA ω-3	9.06 ± 2.27	8.39 ± 2.02	6.71 ± 1.70	10.70 ± 1.59 ^#^	*0.044*
ΣPUFA ω-6	31.85 ± 1.50	34.41 ± 2.54	34.63 ± 2.18	30.16 ± 2.13	0.532
ω-6/ω-3	3.51 ± 0.04	4.10 ± 0.12	5.16 ± 0.03	2.18 ± 0.07 ^#^	*0.036*

The statics significant comparisons are demonstrated by the symbols added to the table: # baseline versus post-treatment; $ placebo versus juçara group. These variables were compared by two-way ANOVA and Bonferroni post hoc test.

**Table 6:** Multiple linear regression analysis of the monocytes MeCP2 mRNA levels with juçara treatment and with oleic fatty acid serum concentration.

MeCP2 mRNA					
	R^2^	β	p	95% CL	VIF
With Juçara ^§^	0.362	0.602	0.014	0.178 to 1.304	1.000
Oleic fatty acid (C18:1n9)	0.410	0.640	0.008	0.013 to 0.071	1.000

CL: Confidence limits; data were; confounders of gender, age and treatment corrected the data. § Juçara is a categorical variable; VIF: variance inflation factor.

## References

[1] Darnton-Hill I., Nishida C., James W. (2007). A life course approach to diet, nutrition and the prevention of chronic diseases. Public Health Nutr..

[2] Delarue J., Magnan C. (2007). Free fatty acids and insulin resistance. Curr. Opin. Clin. Nutr. Metab. Care.

[3] Woods S.C., Seeley R.J., Rushing P.A., D’Alessio D., Tso P. (2003). A controlled high-fat diet induces an obese syndrome in rats. J. Nutr..

[4] Burdge G.C., Lillycrop K.A. (2014). Fatty acids and epigenetics. Curr. Opin. Clin. Nutr. Metab. Care.

[5] Alfaradhi M.Z., Ozanne S.E. (2011). Developmental programming in response to maternal overnutrition. Front. Genet..

[6] Li M., Sloboda D.M., Vickers M.H. (2011). Maternal obesity and developmental programming of metabolic disorders in offspring: Evidence from animal models. Exp. Diabetes Res..

[7] Seki Y., Williams L., Vuguin P.M., Charron M.J. (2012). Minireview: Epigenetic Programming of Diabetes and Obesity: Animal Models. Endocrinology.

[8] Burdge G.C., Hanson M.A., Slater-Jefferies J.L., Lillycrop K.A. (2007). Epigenetic regulation of transcription: A mechanism for inducing variations in phenotype (fetal programming) by differences in nutrition during early life?. Br. J. Nutr..

[9] Lee W., Lee S.Y., Son Y.-J., Yun J.-M. (2015). Gallic Acid Decreases Inflammatory Cytokine Secretion Through Histone Acetyltransferase/Histone Deacetylase Regulation in High Glucose-Induced Human Monocytes. J. Med. Food.

[10] Simar D., Versteyhe S., Donkin I., Liu J., Hesson L., Nylander V., Fossum A., Barrès R. (2014). DNA methylation is altered in B and NK lymphocytes in obese and type 2 diabetic human. Metab. Clin. Exp..

[11] Zhang F.F., Cardarelli R., Carroll J., Fulda K.G., Kaur M., Gonzalez K., Vishwanatha J.K., Santella R.M., Morabia A. (2011). Significant differences in global genomic DNA methylation by gender and race/ethnicity in peripheral blood. Epigenetics.

[12] De Santa F., Narang V., Yap Z.H., Tusi B.K., Burgold T., Austenaa L., Bucci G., Caganova M., Notarbartolo S., Casola S. (2009). Jmjd3 contributes to the control of gene expression in LPS-activated macrophages. EMBO J..

[13] Yan Q., Sun L., Zhu Z., Wang L., Li S., Ye R.D. (2014). Jmjd3-mediated epigenetic regulation of inflammatory cytokine gene expression in serum amyloid A-stimulated macrophages. Cell. Signal..

[14] Rupasinghe H.P.V., Sekhon-Loodu S., Mantso T., Panayiotidis M.I. (2016). Phytochemicals in regulating fatty acid β-oxidation: Potential underlying mechanisms and their involvement in obesity and weight loss. Pharmacol. Ther..

[15] Silva-Martínez G.A., Rodríguez-Ríos D., Alvarado-Caudillo Y., Vaquero A., Esteller M., Carmona F.J., Moran S., Nielsen F.C., Wickström-Lindholm M., Wrobel K. (2016). Arachidonic and oleic acid exert distinct effects on the DNA methylome. Epigenetics.

[16] Schulz M., da Silva Campelo Borges G., Gonzaga L.V., Oliveira Costa A.C., Fett R. (2016). Juçara fruit (Euterpe edulis Mart.): Sustainable exploitation of a source of bioactive compounds. Food Res. Int..

[17] Silva P., Carmo L., Silva G., Silveira-diniz M., Casemiro R., Spoto M. (2013). Physical, Chemical, and Lipid Composition of Juçara (Euterpe Edulis Mart.) Pulp. Braz. J. Food Nutr..

[18] Silva N.A.D., Rodrigues E., Mercadante A.Z., De Rosso V.V. (2014). Phenolic compounds and carotenoids from four fruits native from the Brazilian Atlantic forest. J. Agric. Food Chem..

[19] Oyama L.M., Silva F.P., Carnier J., De Miranda D.A., Santamarina A.B., Ribeiro E.B., Oller Do Nascimento C.M., De Rosso V.V. (2016). Jucąra pulp supplementation improves glucose tolerance in mice. Diabetol. Metab. Syndr..

[20] Jamar G., Santamarina A.B., Mennitti L.V., Cesar H.d.C., Oyama L.M., de Rosso V.V., Pisani L.P. (2018). Bifidobacterium spp. reshaping in the gut microbiota by low dose of juçara supplementation and hypothalamic insulin resistance in Wistar rats. J. Funct. Foods.

[21] Santamarina A., Jamar G., Mennitti L., de Rosso V., Cesar H., Oyama L., Pisani L. (2018). The Use of Juçara (Euterpe edulis Mart.) Supplementation for Suppression of NF-κB Pathway in the Hypothalamus after High-Fat Diet in Wistar Rats. Molecules.

[22] Faul F., Erdfelder E., Lang A.G., Buchner A. (2007). G*Power 3: A flexible statistical power analysis program for the social, behavioral, and biomedical sciences. Behav. Res. Methods.

[23] Campión J., Milagro F.I., Goyenechea E., Martínez J.A. (2009). TNF-α promoter methylation as a predictive biomarker for weight-loss response. Obesity.

[24] Caris A.V., Lira F.S., de Mello M.T., Oyama L.M., dos Santos R.V.T. (2014). Carbohydrate and glutamine supplementation modulates the Th1/Th2 balance after exercise performed at a simulated altitude of 4500 m. Nutrition.

[25] Hardy O.T., Kim A., Ciccarelli C., Hayman L.L., Wiecha J. (2013). Monocytes is a Feature of Metabolic Syndrome in Adolescents. Pediatr. Obes..

[26] Jialal I., Kaur H., Devaraj S. (2014). Toll-like receptor status in obesity and metabolic syndrome: A translational perspective. J. Clin. Endocrinol. Metab..

[27] Wan Z., Durrer C., Mah D., Simtchouk S., Little J.P. (2014). One-week high-fat diet leads to reduced toll-like receptor 2 expression and function in young healthy men. Nutr. Res..

[28] Weiterer S., Uhle F., Lichtenstern C., Siegler B.H., Bhuju S., Jarek M., Bartkuhn M., Weigand M.A. (2015). Sepsis induces specific changes in histone modification patterns in human monocytes. PLoS ONE.

[29] WHO (2000). Obesity: Preventing and Managing the Global Epidemic. Report of a WHO Consultation.

[30] Kamimura M., Baxmann A., Sampaio L., Cuppari L. (2007). Avaliação Nutricional. Nutrição Clínica no Adulto. Guias de Medicina Ambulatorial e Hospitalar.

[31] Alberti K., Zimmet P., Shaw J. (2005). IDF Epidemiology Task Force Consensus Group. The metabolic syndrome: A new worldwide definition. Lancet.

[32] Haun D.R., Pitanga F.J.G., Lessa I. (2009). Razão cintura/estatura comparado a outros indicadores antropométricos de obesidade como preditor de risco coronariano elevado. Revista da Associação Médica Brasileira.

[33] Argentato P.P., Morais C.A., Santamarina A.B., de Cássia César H., Estadella D., de Rosso V.V., Pisani L.P. (2017). Jussara (Euterpe edulis Mart.) supplementation during pregnancy and lactation modulates UCP-1 and inflammation biomarkers induced by trans-fatty acids in the brown adipose tissue of offspring. Clin. Nutr. Exp..

[34] Morais C.A., Oyama L.M., de Moura Conrado R., de Rosso V.V., do Nascimento C.O., Pisani L.P. (2015). Polyphenols-rich fruit in maternal diet modulates inflammatory markers and the gut microbiota and improves colonic expression of ZO-1 in offspring. Food Res. Int..

[35] Christie W. (1989). Chromatpgraphy and Lipids: A Practical Guide.

[36] Livak K.J., Schmittgen T.D. (2001). Analysis of relative gene expression data using real-time quantitative PCR and the 2-ΔΔCT method. Methods.

[37] Jamar G., Pisani L.P., Oyama L.M., Belote C., Masquio D.C.L., Furuya V.A., Carvalho-Ferreira J.P., Andrade-Silva S.G., Dâmaso A.R., Caranti D.A. (2013). Is the neck circumference an emergent predictor for inflammatory status in obese adults?. Int. J. Clin. Pract..

[38] Masquio D.C.L., de Piano A., Campos R.M.S., Sanches P.L., Corgosinho F.C., Carnier J., Oyama L.M., do Nascimento C.M.P.O., de Mello M.T., Tufik S., Dâmaso A.R. (2014). Saturated fatty acid intake can influence increase in plasminogen activator inhibitor-1 in obese adolescents. Horm. Metab. Res..

[39] Santos R., Gagliardi A., Xavier H., Magnoni C., Cassani R., Lottenberg A., Casella Filho A., Araújo D., Cesena F., Alves R. (2013). I Diretriz sobre o consumo de Gorduras e Saúde Cardiovascular. Arquivos Brasileiros de Cardiologia.

[40] Liou Y.A., King D.J., Zibrik D., Innis S.M. (2007). Decreasing linoleic acid with constant α-linolenic acid in dietary fats increases (n-3) eicosapentaenoic acid in plasma phospholipids in healthy men. J. Nutr..

[41] Babio N., Bulló M., Salas-Salvadó J. (2009). Mediterranean diet and metabolic syndrome: The evidence. Public Health Nutr..

[42] Naranjo M.C., Bermudez B., Garcia I., Lopez S., Abia R., Muriana F.J.G., Montserrat-De La Paz S. (2017). Dietary fatty acids on aortic root calcification in mice with metabolic syndrome. Food Funct..

[43] Razquin C., Martinez J.A., Martinez-Gonzalez M.A., Mitjavila M.T., Estruch R., Marti A. (2009). A 3 years follow-up of a Mediterranean diet rich in virgin olive oil is associated with high plasma antioxidant capacity and reduced body weight gain. Eur. J. Clin. Nutr..

[44] Riccardi G., Vaccaro O., Costabile G., Rivellese A.A. (2016). How Well Can We Control Dyslipidemias Through Lifestyle Modifications?. Curr. Cardiol. Rep..

[45] Institute of Medicine of the National Academies (2002). Dietary Reference Intakes for Energy, Carbohydrates, Fiber, Fat, Protein and Amino Acids.

[46] Cintra D.E., Ropelle E.R., Moraes J.C., Pauli J.R., Morari J., de Souza C.T., Grimaldi R., Stahl M., Carvalheira J.B., Saad M.J. (2012). Unsaturated fatty acids revert diet-induced hypothalamic inflammation in obesity. PLoS ONE.

[47] Shimpukade B., Hudson B.D., Hovgaard C.K., Milligan G., Ulven T. (2012). Discovery of a potent and selective GPR120 agonist. J. Med. Chem..

[48] Pan M.-H., Lai C.-S., Wu J.-C., Ho C.-T. (2013). Epigenetic and disease targets by polyphenols. Curr. Pharm. Des..

[49] Shirakawa J., Fujii H., Ohnuma K., Sato K., Ito Y., Kaji M., Sakamoto E., Koganei M., Sasaki H., Nagashima Y. (2011). Diet-induced adipose tissue inflammation and liver steatosis are prevented by DPP-4 inhibition in diabetic mice. Diabetes.

[50] Oliveira V., Marinho R., Vitorino D., Santos G.A., Moraes J.C., Dragano N., Sartori-Cintra A., Pereira L., Catharino R.R., Da Silva A.S.R. (2015). Diets containing alfa-linolenic (omega-3) or oleic (omega-9) fatty acids rescues obese mice from insulin resistance. Endocrinology.

[51] Ju J.-H., Yoon H.-S., Park H.-J., Kim M.-Y., Shin H.-K., Park K.-Y., Yang J.-O., Sohn M.-S., Do M.-S. (2011). Anti-Obesity and Antioxidative Effects of Purple Sweet Potato Extract in 3T3-L1 Adipocytes In Vitro. J. Med. Food.

[52] Garcia-Diaz D.F., Johnson M.H., de Mejia E.G. (2014). Anthocyanins from Fermented Berry Beverages Inhibit Inflammation-Related Adiposity Response In Vitro. J. Med. Food.

[53] Xu W., Zhou Q., Yao Y., Li X., Zhang J., Su G., Deng A. (2016). Inhibitory effect of Gardenblue blueberry (Vaccinium ashei Reade) anthocyanin extracts on lipopolysaccharide-stimulated. J. Zhejiang Univ. Sci. B.

[54] Mena P., Domínguez-Perles R., Gironés-Vilaplana A., Baenas N., García-Viguera C., Villaño D. (2014). Flavan-3-ols, anthocyanins, and inflammation. IUBMB Life.

[55] Lee Y.M., Yoon Y., Yoon H., Park H.M., Song S., Yeum K.J. (2017). Dietary anthocyanins against obesity and inflammation. Nutrients.

[56] Taylor E.M., Jones A.D., Henagan T.M. (2014). A Review of Mitochondrial-derived Fatty Acids in Epigenetic Regulation of Obesity and Type 2 Diabetes. J. Nutr. Health Food Sci..

[57] Lyko F. (2018). The DNA methyltransferase family: A versatile toolkit for epigenetic regulation. Nat. Rev. Genet..

